# Cryo-EM structure of a catalytic amyloid fibril

**DOI:** 10.1038/s41598-023-30711-y

**Published:** 2023-03-11

**Authors:** Thomas Heerde, Akanksha Bansal, Matthias Schmidt, Marcus Fändrich

**Affiliations:** Institute of Protein Biochemistry, Ulm University, 89081 Ulm, Germany

**Keywords:** Cryoelectron microscopy, Biomaterials - proteins, Biocatalysis, Peptides, Prions

## Abstract

Catalytic amyloid fibrils are novel types of bioinspired, functional materials that combine the chemical and mechanical robustness of amyloids with the ability to catalyze a certain chemical reaction. In this study we used cryo-electron microcopy to analyze the amyloid fibril structure and the catalytic center of amyloid fibrils that hydrolyze ester bonds. Our findings show that catalytic amyloid fibrils are polymorphic and consist of similarly structured, zipper-like building blocks that consist of mated cross-β sheets. These building blocks define the fibril core, which is decorated by a peripheral leaflet of peptide molecules. The observed structural arrangement differs from previously described catalytic amyloid fibrils and yielded a new model of the catalytic center.

## Introduction

Amyloid fibrils are linear polypeptide aggregates that were initially identified in the context of debilitating human conditions, such as systemic amyloidosis or neurodegenerative diseases^1,2^. The fibrils represent highly ordered structures that consist of a repetitive pattern of β-strands running perpendicular to the fibril main axis^3^. Several studies showed that amyloid fibrils are able to withstand strongly denaturing conditions, such as high temperatures^4,5^, salt concentrations^6^, pressures^4,5,7^ or extreme pH values^6^. These structural features raised interest in the use of amyloid fibrils as novel types of functional materials. Indeed, amyloid fibrils have been explored with respect to a number of different biotechnological applications, including their use as conductive nanowires^8^, aerogels for water purification^9^, materials for the selective capture of carbon dioxide^10^, agents that deliver genetic material into cells^11^, as well as catalytic agents^12–18^.

That amyloid fibril can present catalytic activities was found, for example, for Cu^2+^ binding Aβ(1-42) amyloid fibrils from Alzheimer’s disease that catalyze Fenton reactions and generate H_2_O_2_ or other reactive oxygen species^19,20^. The use of Zn^2+^ ions in biological enzymes inspired the rational design of Zn^2+^-dependent catalytic centers in self-assembling peptide systems, such as those formed by the peptides acetyl–IHIHIQI–amide, acetyl-IHIHIYI-amide and acetyl-IHVHLQI-amide^5,12,14,15^. Their amyloid structures bind Zn^2+^ ions and convert the substrate p-nitrophenyl acetate into nitrophenolate and acetate^5,12,14,15^. A related ester hydrolyzing activity was reported for the Mn^2+^-bound fibrils of the peptides acetyl-NADFDGFQMAVHV-amide and acetyl-SDIDVFI-amide that resemble natural adenosine triphosphatase in their ability to hydrolyze phosphodiester bonds^16,18^. In addition, Cu^2+^-bound acetyl-IHIHIYI-amide fibrils hydrolyze the phosphoester bond of paraoxon, a known organophosphate pesticide^17^. Ester hydrolytic activities are not necessarily dependent on the presence of transition metal ions and amyloid fibrils formed from the peptide of HSGQQKFQFQFEQQ catalyze this reaction without the need of a transition metal ion^13^.

In this study we have used cryo electron microscopy (cryo-EM) to analyze the molecular structure of the catalytic amyloid fibril formed by the peptide acetyl-LHLHLRL-amide. We find that the peptide self-assembles into polymorphic spectrum of amyloid fibrils that consist of similarly shaped building blocks or fibril protofilaments (PFs). Each PF is formed by two paired cross-β sheets that interact through the hydrophobic side of the amphipathic peptide, while the peptide’s histidine residues are pointing outwards. The fibril PFs are partially decorated by an outer layer of peptides, which may form, together with the peptides in the fibril core, the catalytically active zinc binding site.

## Results and discussion

### Formation of catalytic amyloid fibrils

We incubated three peptides (acetyl-IHIHIQI-amide, acetyl-IHVHLQI-amide, acetyl-LHLHLRL-amide), which were previously reported to form catalytic amyloid fibrils^12,15^, for 3 days at 22 °C in 25 mM tris(hydroxymethyl)aminomethane (Tris) buffer, pH 8.0, containing 1 mM ZnCl_2_. Transmission electron microscopy (TEM) revealed the presence of aggregates in all samples at the end of the incubation period (SI Fig. 1), but the best morphological properties in terms of an elongated structure and twisted helical symmetry were obtained with the peptide acetyl-LHLHLRL-amide (SI Fig. 1). This sample contained substantial amounts of relatively straight and long fibrils that showed several well-resolved cross-overs.

To confirm the catalytic activity of the acetyl-LHLHLRL-amide fibrils, we monitored the time-dependent absorbance (at 348 nm and 405 nm) of the product p-nitrophenol that is formed by hydrolysis of the substrate p-nitrophenyl acetate (SI Fig. 2). Fitting the Michaelis–Menten plot allowed us to obtain the Michaelis constant K_M_ (1.47 ± 0.19 mM at 348 nm and 1.44 ± 0.08 mM at 405 nm) and the turnover number *k*_cat_ (2.01 ± 0.22 × 10^–2^ s^–1^ at 348 nm and 2.27 ± 0.16 × 10^–2^ s^–1^ at 405 nm). The ratio of the two, expressed as the parameter *k*_cat_/K_M_, indicates the catalytic efficiency of the enzymatic reaction. Our values of 13.7 ± 0.5 M^–1^ s^–1^ at 348 nm and 15.7 ± 0.4 M^–1^ s^–1^ at 405 nm indicate a modest catalytic efficiency that is much lower than the so-called diffusion limit of enzymatic reactions, which lies within the range of 10^8^ or 10^9^ M^–1^ s^–1^.

The obtained values for K_M,_ *k*_cat_ and their ratio are similar to the values measured with other transition metal-binding amyloids, where K_M_ ranges from 0.02 to 1.9 mM, *k*_cat_ from 0.27 × 10^–2^ to 5.59 × 10^–2^ s^–1^; *k*_cat_/K_M_: 10^1^ to 3.5 × 10^2^ M^–1^ s^–1^^12,14,15^. The K_M_ and *k*_cat_/K_M_ values also relate to the values obtained with natural enzymes, such as carbonic anhydrase, α-chymotrypsin, carboxylesterase and acetylcholinesterase which range in the case of *k*_cat_/K_M_ from 9 × 10^1^ to 5 × 10^6^ M^−1^ s^−1^, and in case of K_M_ from 5.9 mM to 5.2 × 10^5^ M for the hydrolysis of p-nitrophenyl acetate^21–24^. Only the turnover number *k*_cat_ is higher in the globularly folded enzymes (2.3 s^−1^ to 4.7 × 10^3^ s^−1^)^21–24^ than in catalytic amyloids.

**Figure 1:**
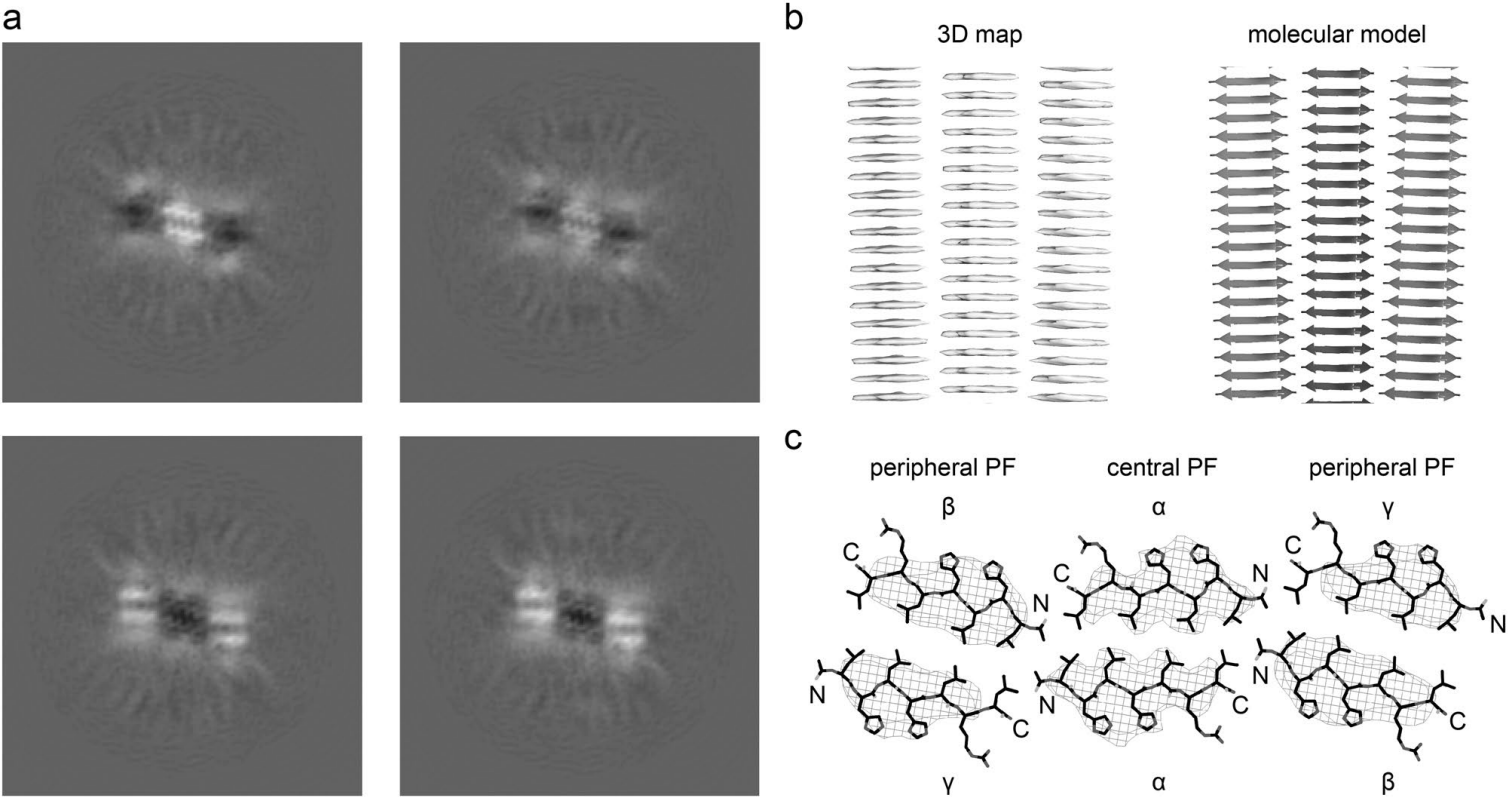
Cryo-EM structure of the catalytic amyloid fibril of peptide acetyl-LHLHLRL-amide. (**a**) 1.04 Å thick slices of the reconstructed 3D map at different z-axial positions. (**b**) Side views of the 3D map (left) and of the molecular model (right, ribbon diagram, PDB entry 8B3A). (**c**) Cross-sectional view of one molecular layer of the reconstructed density (wireframe lattice), superimposed with the molecular model (stick representation). The peptide adopts three topological distinct positions (α, β, γ) in the fibril.

Based on cryo-EM we could discern several fibril morphologies that varied in the fibril width and in the distance between adjacent cross-overs (SI Fig. 3a). Although the fibrils were often decorated with peripherally attached peptide oligomers (SI Fig. 3b), we could distinguish three relatively abundant fibril morphologies (SI Fig. 4), which we term here morphologies I, II and III in the order of their ascending width (SI Fig. 5). Reconstruction of the 3D maps of these fibrils was attempted, but only morphology I allowed us to obtain a reconstruction with a reasonable nominal resolution of 3.78 Å (Supplementary Table 1), based on 0.143 Fourier shell correlation (FSC) criterion (SI Fig. 6a). The fibril is polar and C2 symmetrical. Its 3D map is better defined in the direction of the fibril z-axis than in the fibril cross-section (Fig. 1a, b), and allowed us to model the fibril structure with a model resolution of 3.9 Å (SI Fig. 6b, Supplementary Table 2). Projections of the model show good correspondence to the two-dimensional class averages (SI Fig. 6c).

### Packing of the peptides and zipper-like structure of the fibril

**Figure 2:**
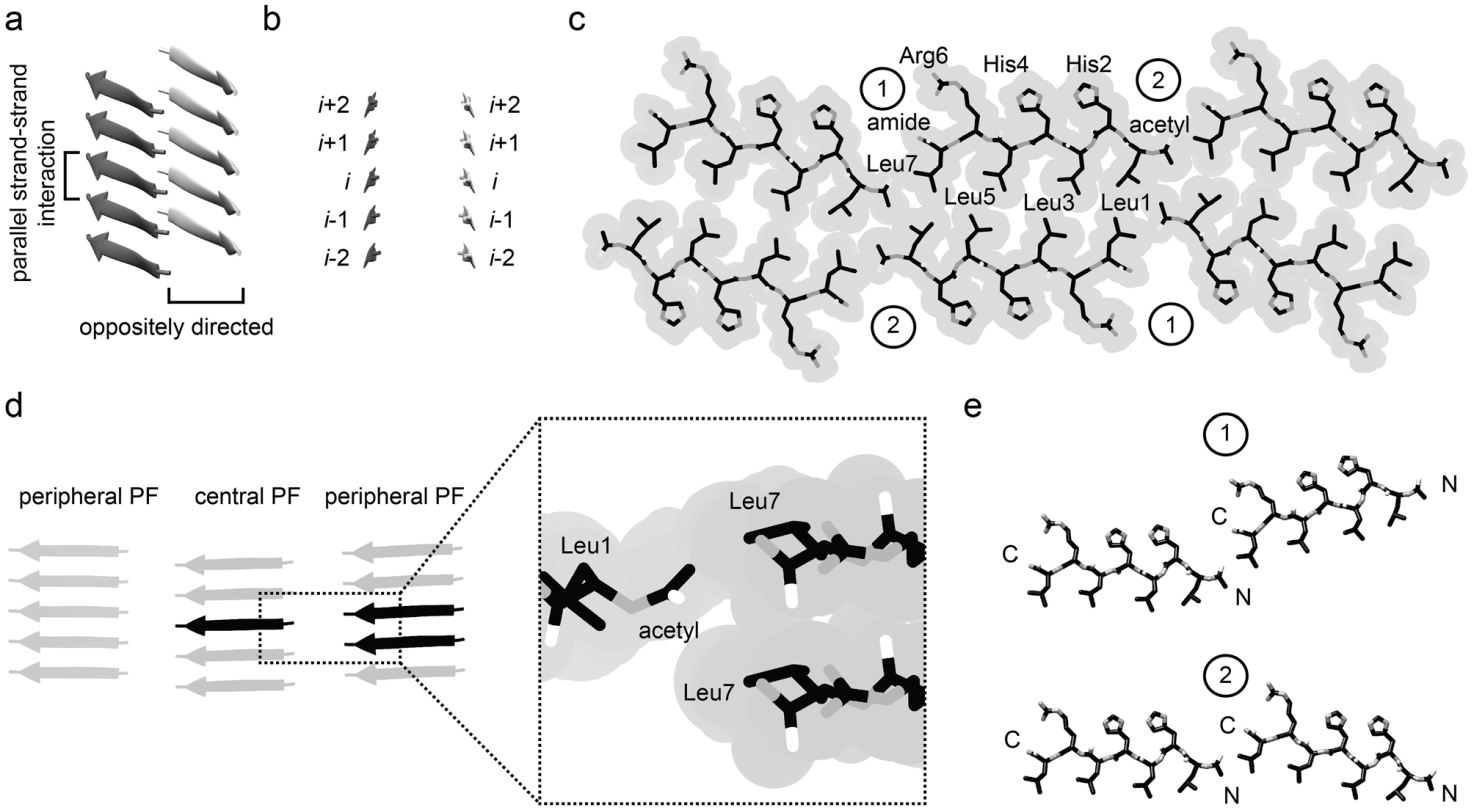
Packing of the peptides in the fibril. (**a**) Ribbon diagram of five molecular layers of the central PF. Parallel strand-strand interactions occur within the same β-sheet. Strands in the same molecular layer of the two β-sheets are oppositely directed. (**b**) Ribbon diagram of layers i – 1 to i + 1 of the central PF, viewed along the strands. The two sheets are not staggered relative to one another. (**c**) Cross-sectional view of a stick model of one molecular layer of the fibril, superimposed with the outline of the van der Waals surface (grey). (**d**) Side view of the proximal β-sheets of the three PFs, showing the staggering of adjacent PFs. Close up: Contact site of two PFs. (**e**) There are two peptide-peptide contact modes (1 and 2) as labelled in panel (**c**).

The fibril core contains three similarly shaped building blocks, which we refer to as the fibril PFs (Fig. 1c). There is one central PF and two peripheral PFs. Each PF consists of a face-to-face packed pair of mated cross-β sheets (Fig. 2a). The PF structure resembles the structure of β-sheet zippers seen in certain peptide crystals^25^. The strands of the same β-sheet present a parallel orientation relative to one another, while they are oppositely directed to the strands in the other sheet of the PF (Fig. 2a). The two peptide stacks from the same PF are not staggered relative to one another (Fig. 2b). Their interface is hydrophobic and formed by the side chains of the peptide’s four leucine residues (Leu1, Leu3, Leu5 and Leu7). The packing of these residues is tighter in the central PF than in the peripheral PFs (Fig. 2c), suggesting that the PF structure can adapt to the exact position of the PF in the fibril topology. Adjacent PFs are staggered in the direction of the fibril z-axis so that each molecular layer of the central PF interacts with two molecular layers of the peripheral PFs, and vice versa (Fig. 2d). The interface between two PFs is hydrophobic and formed by the side chain of Leu7 and the methylene group of the N-terminal acetyl modification (Fig. 2c). The peptides show, within the fibril cross-section, two types of kinked head-to-tail interactions (Fig. 2e). The two types of contact models across the PFs result in three non-equal peptide positions in fibril (Fig. 1c). These data further corroborate the structural plasticity in the packing of these amyloid fibrils.

### Comparison with a previously reported fibril structure

**Figure 3:**
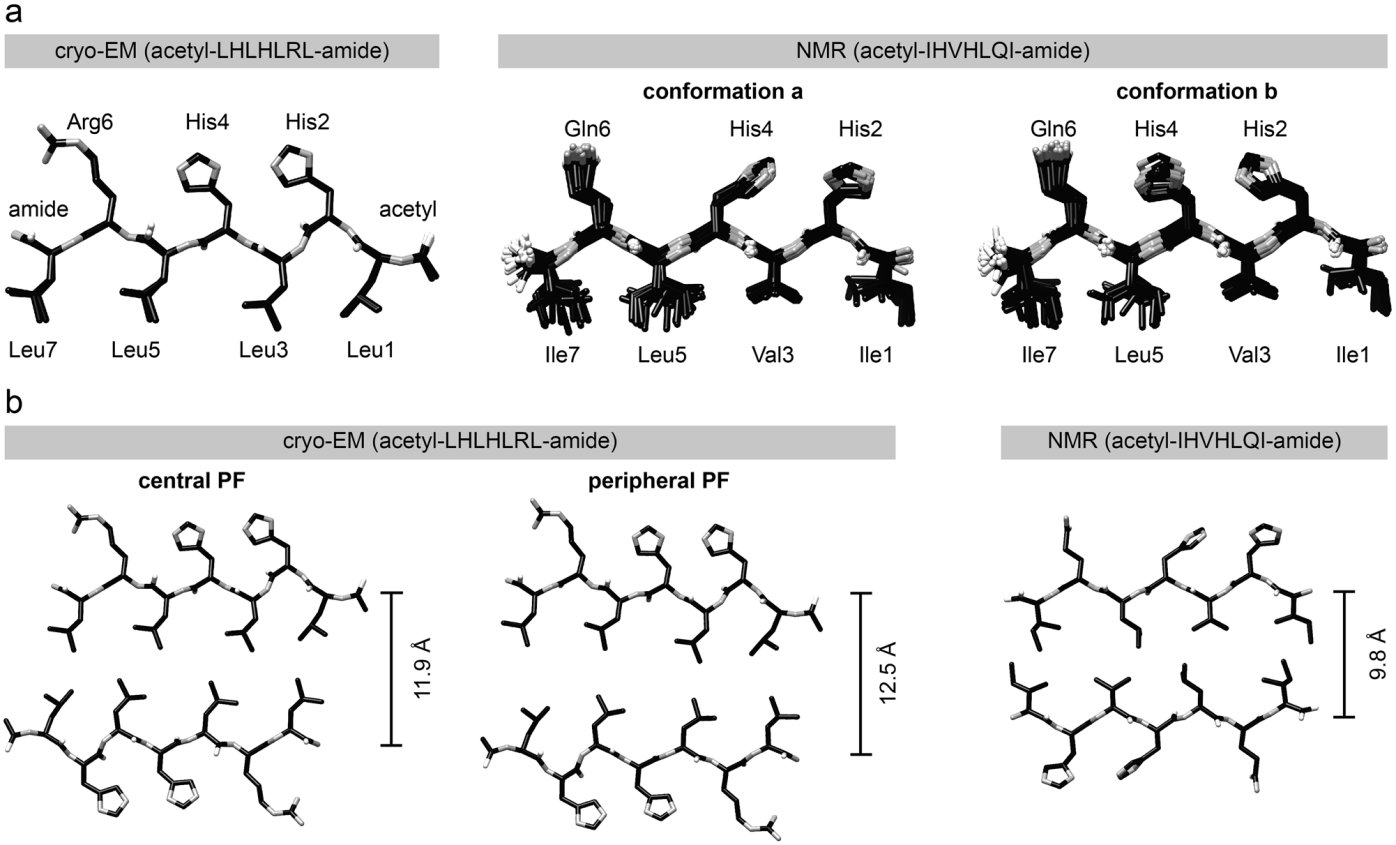
Comparison of our structure with previous data. (**a**) Left: superimposition of the three (closely similar) peptide conformations (α, β, γ, see Fig. 1c) of peptide acetyl-LHLHLRL-amide in our structure. Center and right: superimpositions of families of 20 NMR structures of type a and b conformations of the peptide acetyl-IHVHLQI-amide. The NMR structures are taken from the PDB entry 5UGK^15^, chains A and C. (**b**) Left and center: PF peptide packing in one molecular layer of our structure. Left: central PF, center: peripheral PF. Right: peptide packing in the previous NMR structure^15^.

The backbone conformation in our fibril is similar to two previously described conformations which were obtained with peptide acetyl-IHVHLQI-amide (Fig. 3a) that also forms catalytic amyloid fibrils^15^. These fibrils do not present a clear helical symmetry (SI Fig. 1), and were thus not to be suitable for cryo-EM. Their previous analysis with solid-state NMR spectroscopy^15^ identified two types of conformations (a and b) that differed in the side chain rotamers of residues His2 and His4 (Fig. 3a). The two conformers occurred alternatingly in consecutive layers of the fibril (SI Fig. 7). This assembly resulted in two rows of Zn^2+^ binding sites along each cross-β sheet.

We attempted to reconstruct our fibril by implementing fibril symmetries that would have allowed for two peptide conformations, such as a C2 symmetry with an axial repeat of ~ 9.4 Å or pseudo 2_1_ screw symmetry, in which a ~ 180° rotation is combined with an axial translation of ~ 4.7 Å (SI Fig. 8). However, the resulting 3D maps were not better than the ones obtained with a C2 symmetry and did not provide evidence for different types of peptide conformations in our fibril. The density of our 3D map falls off sharply next to His2 (SI Fig. 9), indicating that this residue (and consequently also residue His4) adopt a b-type conformation in our fibril.

Another difference of our fibril from the previously described structure concerns the packing distance between the mated cross-β sheets. This distance is approximately 12 Å in our fibril and roughly 10 Å in the previous structure (Fig. 3b). Differences in the packing distance reflect the different sizes of the residues at the interface between the two cross-β sheets^15^, which is formed exclusively by leucine residues in our fibril and by isoleucine, leucine and valine residues in the previously reported structure (Fig. 3b).

### The PF core is sandwiched between two outer peptide leaflets

**Figure 4:**
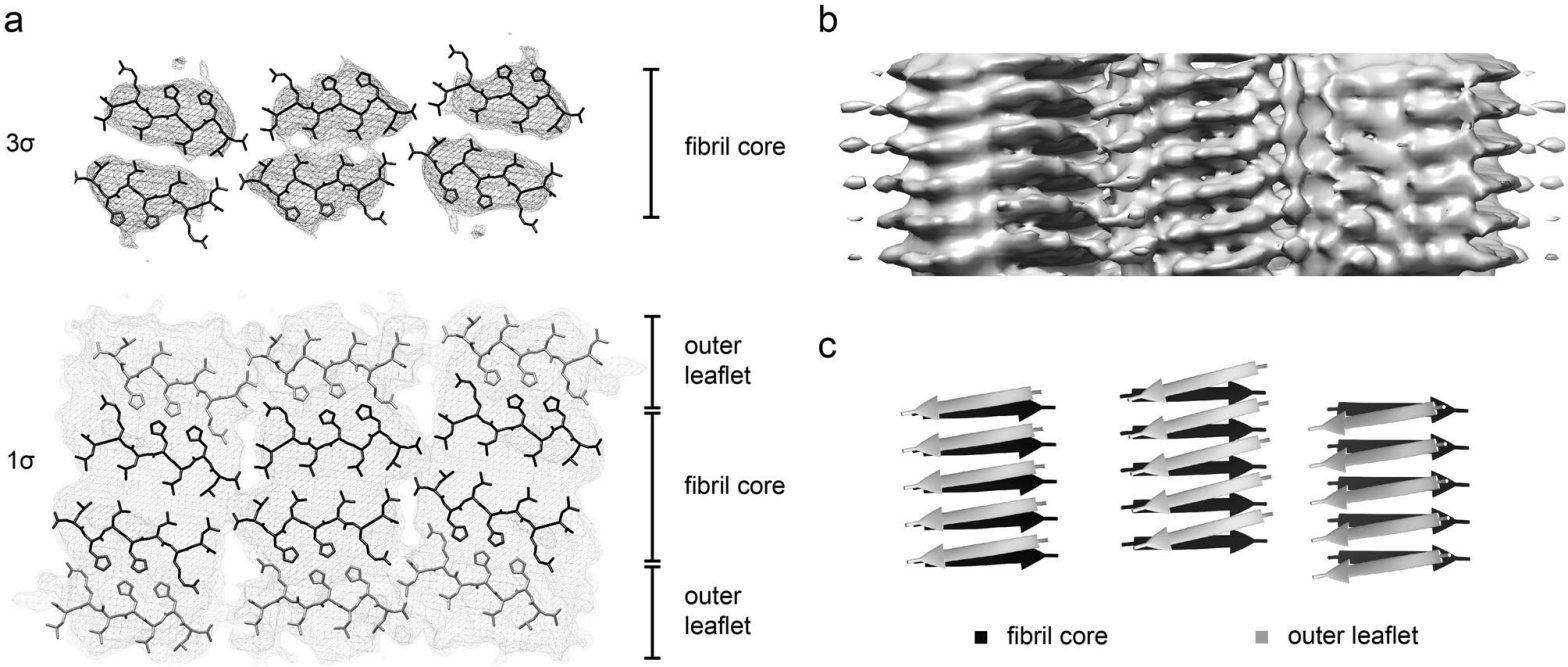
Low density features in the outer peptide leaflet. (**a**) Cross-sectional view of the 3D map, rendered at 1σ and 3σ, superimposed with the model of the fibril core (black sticks) and a possible placement of peptides in the outer leaflet (grey sticks). The peptide conformation in the outer leaflet was taken from the fibril core without further adjustments of its conformation. (**b**) Side view of the reconstructed density map as surface representation to indicate the ~ 4.7 Å z-axial repeat and the tilt of the strands in the outer leaflet. (**c**) Ribbon diagram of the peptides fitted to the outer leaflet (grey arrows) and of the fibril core (black arrows), showing their different tilt.

The 3D map of our fibril contains, in addition to the high-density features of the fibril core, regions of lower density that become evident, if the 3D map is rendered at a peripheral lower threshold (Fig. 4a). These low-density regions indicate the presence of two additional leaflets of peptides on either side of the fibril core. The cross-sectional size of these regions fits six peptides per molecular layer (Fig. 4a). They show a z-axial periodicity of ~ 4.7 Å (Fig. 4b), consistent with peptides being present in a cross-β conformation (Fig. 4c). The strands of these cross β-sheets are tilted by ~ 10° relative to the β-strands of the fibril core, which run almost perpendicular to the fibril axis (Fig. 4c). The diffuse nature of the 3D map within the outer leaflet suggests that the peptides are present in sub-stoichiometric amounts compared with the peptides of the fibril core. That is, they may not be present in every molecular layer (Fig. 4b) and become averaged out in the reconstruction process. While it was not possible to determine the underlying peptide conformations unambiguously, the peptides of the outer leaflet are able to form, together with the peptides in the fibril core, a cluster of four histidine residues (Fig. 4) that occur in the vicinity of the contact points between the differently tilted peptides of the PF core and outer leaflet.

### Structure of the Zn^2+^ binding site

**Figure 5:**
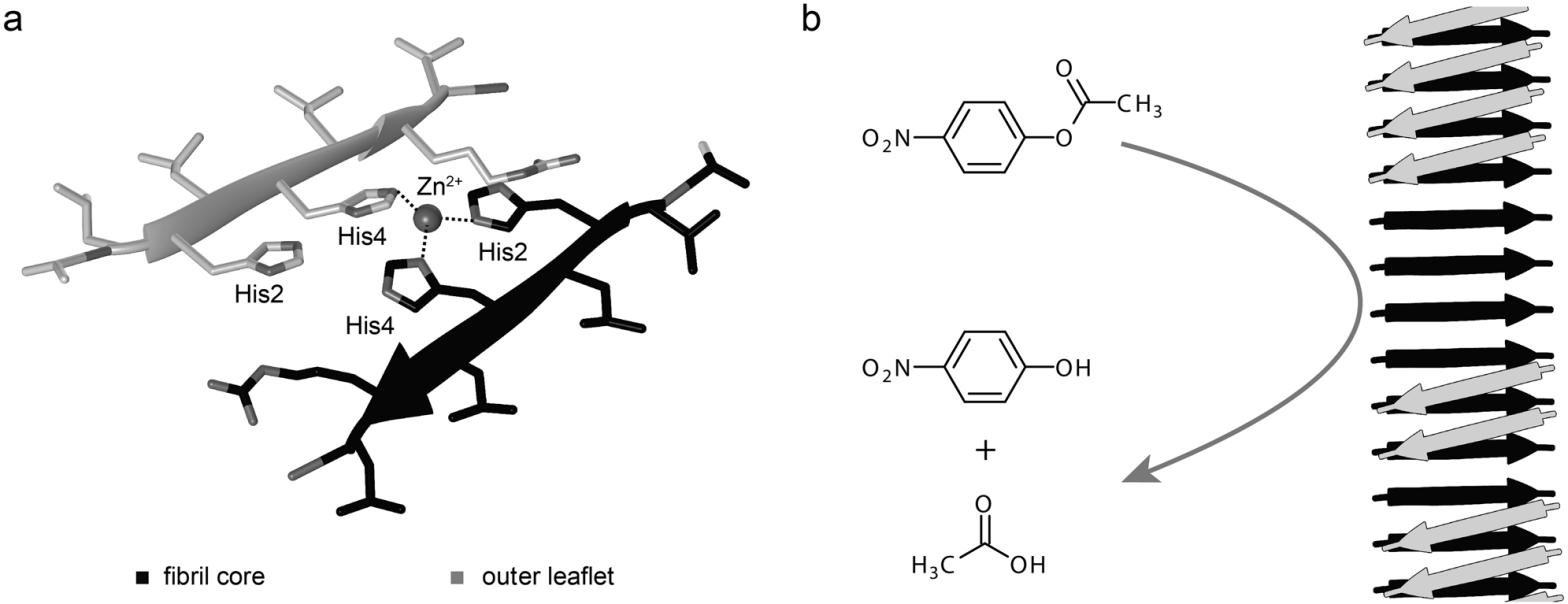
Molecular model of the zinc binding site. (**a**) Molecular arrangement of the Zn^2+^ binding site, formed by a peptide from the fibril core (black) and from the outer leaflet (grey). The distance between the nitrogen atoms of the imidazole groups and the Zn^2+^ ion is, along the dotted lines, approximately 2 Å. The distances between the Zn^2+^ complexing nitrogen atoms are between 3.3 and 3.6 Å. (**b**) Gaps in the outer leaflet of the amyloid fibril allow access of the substrate to the catalytic center.

The histidine residues were designed into the peptide sequence to create a Zn^2+^ binding site^12,14,15^. The design was guided by the histidine-dependent Zn^2+^ bindings sites in catalytically active globular proteins^26,27^. Most of these proteins bind Zn^2+^ ions by a triangular arrangement of three histidine residues or of two histidines and an aspartate or glutamate residue^26,28^. An example hereof is provided in SI Fig. 10a, which shows the catalytic center of the human matrix metalloproteinase-13^29^. The histidine residues of these enzymes bind the Zn^2+^ ion through the imidazole N_ε_ or N_δ_ atoms which occur at a distance of 3–3.5 Å in the protein^26,27^. The intramolecular distance between atom N_ε_ of His2 and atom N_δ_ of His4 is ~ 3.3 Å in our fibril (SI Fig. 10b), which is compatible with the distances of zinc binding nitrogen atoms in natural enzymes, while the distance between the N_ε_ or N_δ_ atoms in adjacent molecular layers of the fibril is too large (~ 4.7 and ~ 5.7 Å, SI Fig. 10b) to be able to contribute to the formation of a catalytically active zinc binding site. These data imply that the catalytic center of our fibril cannot be formed by peptides from two consecutive layers of the fibril and must arise instead from a combination of peptides from the fibril core and from the outer leaflet (Fig. 5a). That is, the catalytic center is sandwiched between two cross β-sheets, one from the fibril core and one in the outer leaflet. Indeed, there is a weak density feature in our 3D map at the position where the zinc ion would be expected (SI Fig. 11). However, the zinc ion is less visible in our 3.78 Å resolution structure than in cryo-EM structures of much higher resolution^30^. An obvious problem arising from this arrangement is that the catalytic center is not solvent exposed and may not be accessed readily by the substrate. To resolve this paradox, we suggest that it could be important for the fibril activity that the β-sheets in the outer peptide leaflet are not continuous but interrupted by gaps (Fig. 5b). These gaps expose the catalytic center and provide access to the substrate binding site so that the catalysis can occur.

### Structure of the catalytic site in different fibril morphologies

This structure of the catalytic center is relevant for other fibril morphologies in the sample as indicated by a comparison of the low-resolution 3D maps of morphologies II and III with the map of morphology I. This comparison reveals similar construction principles in the different filaments if the map of morphology I is filtered to ~ 9 Å resolution to match the resolutions of the 3D maps of morphologies II and III (SI Fig. 12). We find that the cross-section of morphology I is constructed from H-shaped units in which the left and the right bar of the letter H are formed by the two peptides, while the zinc ion and its surrounding histidine residues constitute the letter´s crossbar. Similar H-shaped features can be discerned in both other fibril morphologies so that there are 6 H-shaped density features in the cross-section of fibril morphology I (SI Fig. 12), eight in morphology II and ten in morphology III (SI Fig. 12). We conclude that the polymorphism of fibril morphologies in this sample arises from different numbers of PFs that are packed up side-by-side in a related fashion thereby constituting different but structurally related fibril architectures that share the same basic construction principle of their PFs and of their catalytic centers.

## Conclusion

Analysis of the structure of the catalytic amyloid fibrils formed from acetyl-LHLHLRL-amide peptide revealed that the sample contains several distinct fibril morphologies that are based on a common construction principal. The fibril consists of PFs in which two cross-β sheets are mated in a zipper-like fashion through hydrophobic interactions between the leucine residues, while the histidine residues, which participate in formation of the catalytically active zinc binding site are pointing outwards. This core structure of the fibril is decorated by an outer leaflet of peptides, which forms, together with the peptides in the fibril core, the active site of these catalytic agents. Hence, an alternating arrangement of two peptide conformations within a cross-β sheet, which was found previously for a related peptide^15^, is inconsistent with our fibril and may not explain the active site in our fibril. Instead, we find that peptides from the fibril core and from the outer leaflet may be able to form a catalytically active zinc binding site that is able to explain the observed catalytic activity. The zinc binding site occurs between two cross-β sheets, suggesting that it requires structural gaps within the outer peptide leaflet to allow substrate to enter the active site and to become converted into the reaction products. These data shed new light on the mechanisms of catalytic amyloid fibrils and in the structure of their catalytic site. Such information could be important for future developments to further modulate the activities of catalytic amyloid for potential bionano-technological applications.

## Methods

### Fibril preparation

Purified lyophilized peptides (acetyl-LHLHLRL-amide, acetyl-IHIHIQI-amide, acetyl-IHVHLQI-amide) were obtained at a purity of more than 98% from the Core Unit Peptide-Technologien of the Interdisciplinary Center for Clinical Research Leipzig. The peptides were dissolved at a concentration of 2.5 mg mL^−1^ in 10 mM hydrochloric acid. The exact peptide concentration was then determined spectrophotometrically using a LAMBDA Bio + UV–Vis spectrophotometer (PerkinElmer) and a 105.201.-QS quartz cuvette (Hellma), using the extinction at 214 nm, the Lambert–Beer law and molar extinction coefficients of 17,916 M^−1^ cm^−1^ (acetyl-LHLHLRL-amide), 17,956 M^−1^ cm^−1^ (acetyl-IHIHIQI-amide) or 17,954 M^−1^ cm^−1^ (acetyl-IHVHLQI-amide), which were calculated as described previously^31^. From these initial peptide stocks we prepared the samples at a final peptide concentration of 50 µM in 25 mM Tris at pH 8 and 1 mM ZnCl_2_. The samples were incubated for 3 days at 22 °C.

### Measurement of the catalytic parameters

The catalytic properties were examined based on a previously established protocol^12,32^. Each sample contained 10 µM catalytic amyloid fibrils (their quantification is based on the peptide concentration), 25 mM Tris, pH 8, 1 mM ZnCl_2_, 2% (v/v) acetonitrile and substrate concentrations of 0.2 mM, 0.5 mM, 1 mM, 1.5 mM and 2 mM. These samples were prepared by mixing the following solutions as appropriate: 50 µL enzyme (40 µM acetyl-LHLHLRL-amide, 25 mM Tris, pH 8, 1 mM ZnCl_2_), 146 µL buffer (25 mM Tris, pH 8, 1 mM ZnCl_2_) and 4 µL of the appropriate substrate stock dissolved in acetonitrile. The measurement of the product formation was carried out in a 96-well plate (Greiner Bio-One, 96 F-Bottom) at room temperature using a FLUOstar Omega plate reader (BMG Labtech). We monitored the extinction of the product p-nitrophenol at 348 nm and 405 nm. The measured extinction was converted into the product concentration using a molar extinction coefficient of 3688 M^-1^ at 348 nm and 10,058 M^−1^ at 405 nm that were experimentally determined by calculating the slope of the linear fit of the absorbance of differently concentrated samples of PNP in 25 mM Tris, pH 8, 1 mM ZnCl_2_ at 348 nm and 405 nm. The product formation rates v_0_ were obtained by a linear fit of the product concentration versus time. The K_M_ value was obtained by fitting the plot of v_0_ versus the free substrate concentration [S] to the Michaelis–Menten equation v_0_ = V_max_[S]/(K_M_ + [S]) in which V_max_ is the maximum reaction rate. The turnover number *k*_cat_ was obtained by *k*_cat_ = V_max_/[E]_0_ where [E]_0_ is the total enzyme concentration. *k*_cat_/K_M_ was calculated from the obtained values for *k*_cat_ and K_M_.

### Cryo-EM

A 3.5 µL aliquot of the fibril sample was applied onto a glow-discharged holey carbon coated grid (400 mesh C-flat 1.2/1.3), blotted from both sides with filter paper and plunge-frozen in liquid ethane using a Vitrobot Mark 3 (Thermo Fisher Scientific). The optimization of the grids was done by monitoring the grid quality at 200 kV with a JEM-2100 transmission electron microscope (Jeol) which was equipped with TemCam F-216 (TVIPS). The data set for three-dimensional (3D) reconstruction was collected with a K2-Summit detector (Gatan) that was operated in counting mode. Data acquisition parameters are listed in Supplementary Table 1.

### Helical reconstruction

IMOD^33^ was used to correct the movie frames for the gain reference. Motion correction and dose-weighting was done using MOTIONCOR 2.1^34^. Gctf^35^ was used to estimate the contrast transfer function from the motion-corrected images. Helical reconstruction was carried out by using RELION 3.1^36^. The fibrils were picked manually, and segments were extracted by applying the parameters listed in Supplementary Table 1. A featureless cylinder, which was created by using relion_helix_toolbox, was used as initial 3D model. The resulting reconstructions showed partially separated β-strands in the x–y plane and along the fibril axis that indicated the presence of six identical protein stacks, related by a C2 symmetry. Imposing these symmetries during reconstruction yielded clearly separated β-strands. 3D classification with local optimization of helical twist and rise and a C2 symmetry were used to further improve the model. The best 3D class was finally reconstructed with local optimization of the helical parameters using 3D auto-refinement. A central part of 30% of the intermediate asymmetrical reconstruction was used for all 3D classification and auto-refine processes. The final reconstruction was post-processed with a soft-edge mask and B-factor sharpened. The estimate of the resolution of the reconstruction was carried out based on the FSC at 0.143 between two independently refined half-maps.

### Model building

The model was built de novo using the program Coot^37^. A poly-l-Ala chain was traced along the main chain density that was afterwards mutated according to residues of the peptide sequence. The structure was then manually refined further in Coot. The atomic clashes, rotamer and Ramachandran outliers and model geometry were analyzed by the validation output generated by MolProbity^38^ and the comprehensive validation tool in Phenix^39^. Once a satisfactory fit of the main and side-chain density was achieved for one polypeptide chain, a five layered fibril stack comprising 30 peptide molecules was assembled using the pdbsymm tool implemented in Situs^40^. The described process of iterative refinement and modeling was repeated for the fibril stack over and over again, until the refinement converged to produce reasonable density to model fit. The structural statistics for refinement and model building are listed in Supplementary Table 2.

### Sample statistics

The error bars shown in this manuscript report the standard deviation.

## Supplementary Information

Supplementary Information.

## Data Availability

The reconstructed cryo-EM map was deposited in the Electron Microscopy Data Bank with the accession codes EMD-15824. The coordinates of the fitted atomic model were deposited in the Protein Data Bank (PDB) under the accession code PDB 8B3A. The following previously published coordinates were used in Fig. 3 and SI Fig. 7: PDB 5UGK, and in SI Fig. 10: PDB 1XUD.
