# Supplementary material for: Cryo-EM structure of a catalytic amyloid fibril
Source: Sci Rep. 2023 Mar 11;13:4070. doi: 10.1038/s41598-023-30711-y (PMC10008563; doi:10.1038/s41598-023-30711-y)
Supplement: Supplementary file 1 — Supplementary Information. [file 41598_2023_30711_MOESM1_ESM.pdf]

Supplementary Information

Cryo-EM structure of a catalytic amyloid fibril

Thomas Heerde[a]*, Akanksha Bansal[a], Matthias Schmidt[a], Marcus Fändrich[a]

Affiliations

[a] Institute of Protein Biochemistry, Ulm University, 89081 Ulm, Germany

Correspondence

*Correspondence to: thomas.heerde@uni-ulm.de

Supplementary Figures

Supplementary Figure 1

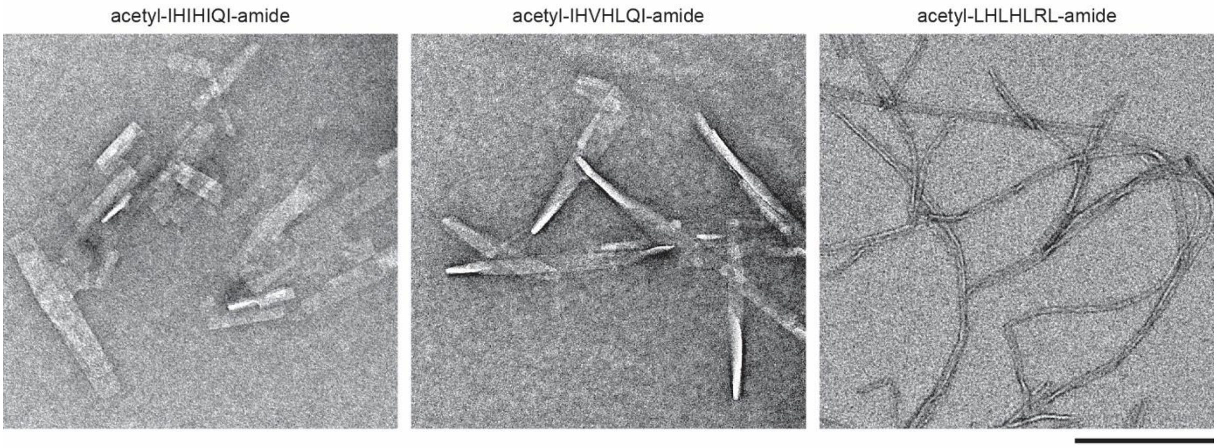

Supplementary Figure 1

TEM images of catalytic amyloid fibrils formed from different peptides.

TEM images of negatively stained fibrils formed from peptides (acetyl-IHIHIQI-amide, acetyl-IHVHLQI-amide, acetyl-LHLHLRL-amide) after 3 days of incubation. Scale bar: 200 nm

Supplementary Figure 2

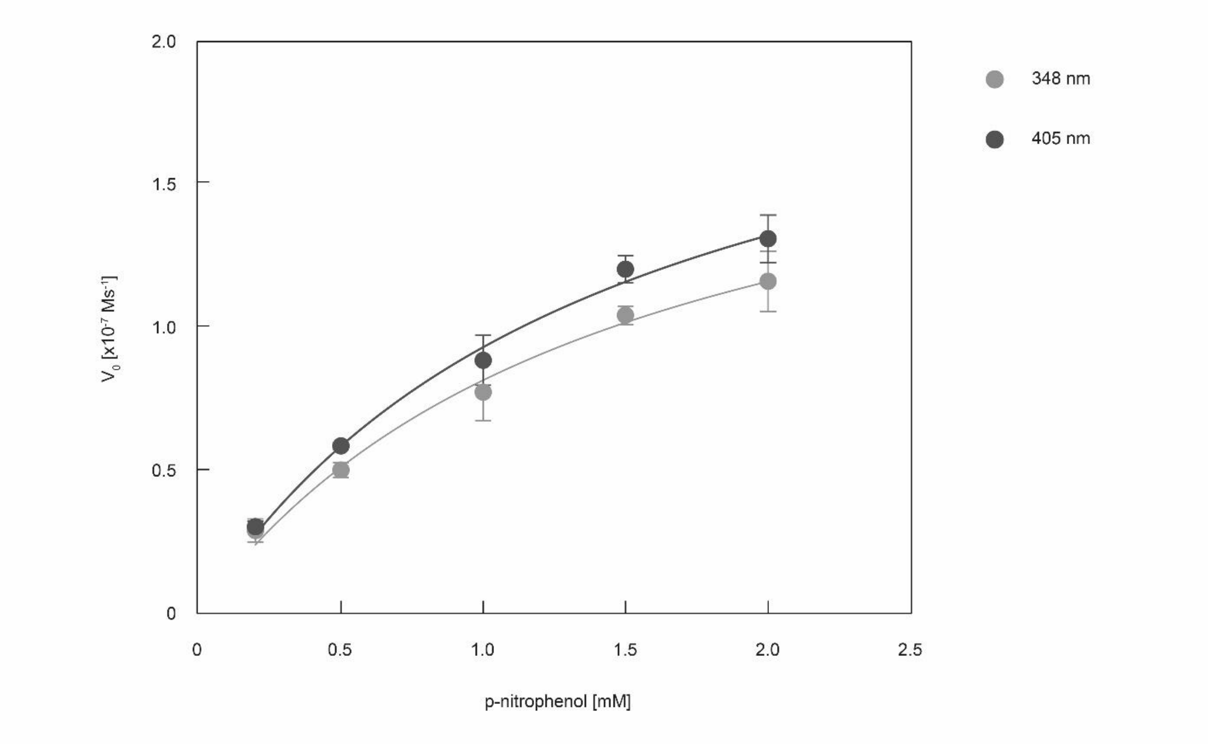

Supplementary Figure 2

Catalytic efficiency of Acetyl-LHLHLRL-amide fibrils

Plot of V_0 versus the p-nitrophenol concentration for the conversion of p-nitrophenyl acetate to p-nitrophenol, measured at 348 nm (light grey) and 405 nm (dark grey) (n = 3).

Supplementary Figure 3

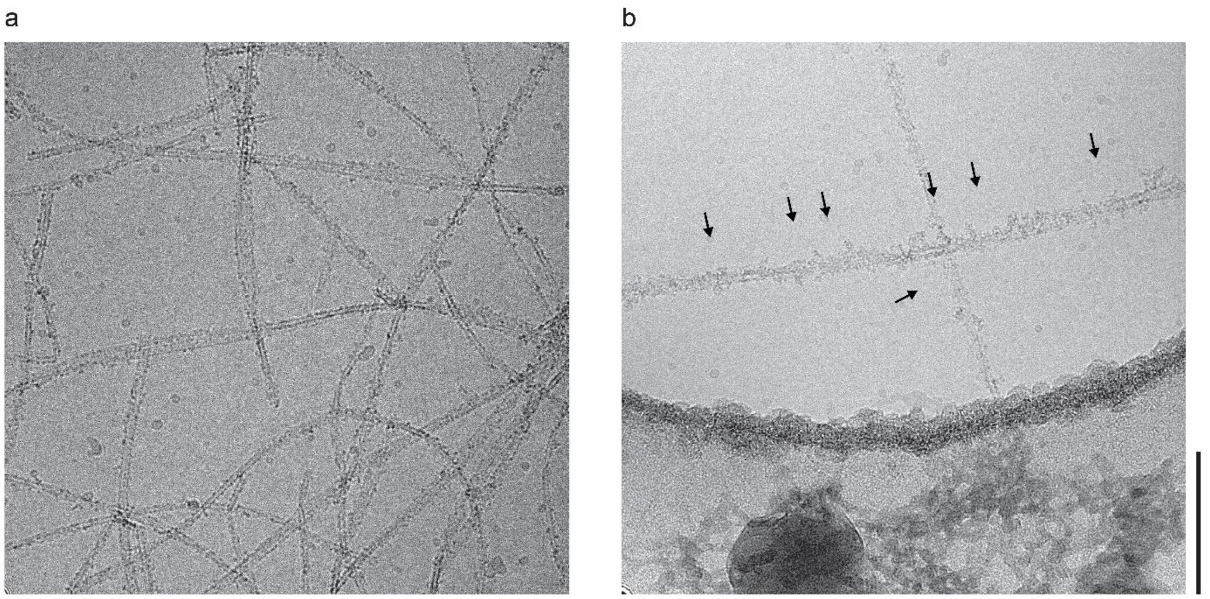

Supplementary Figure 3

Acetyl-LHLHLRL-amide fibril structure viewed by cryo-EM.

(a) Cryo-EM image showing the polymorphism of acetyl-LHLHLRL-amide fibrils. (b) Example of two fibrils with peripherally attached peptide oligomers (arrows). Scale bar: 100 nm.

Supplementary Figure 4

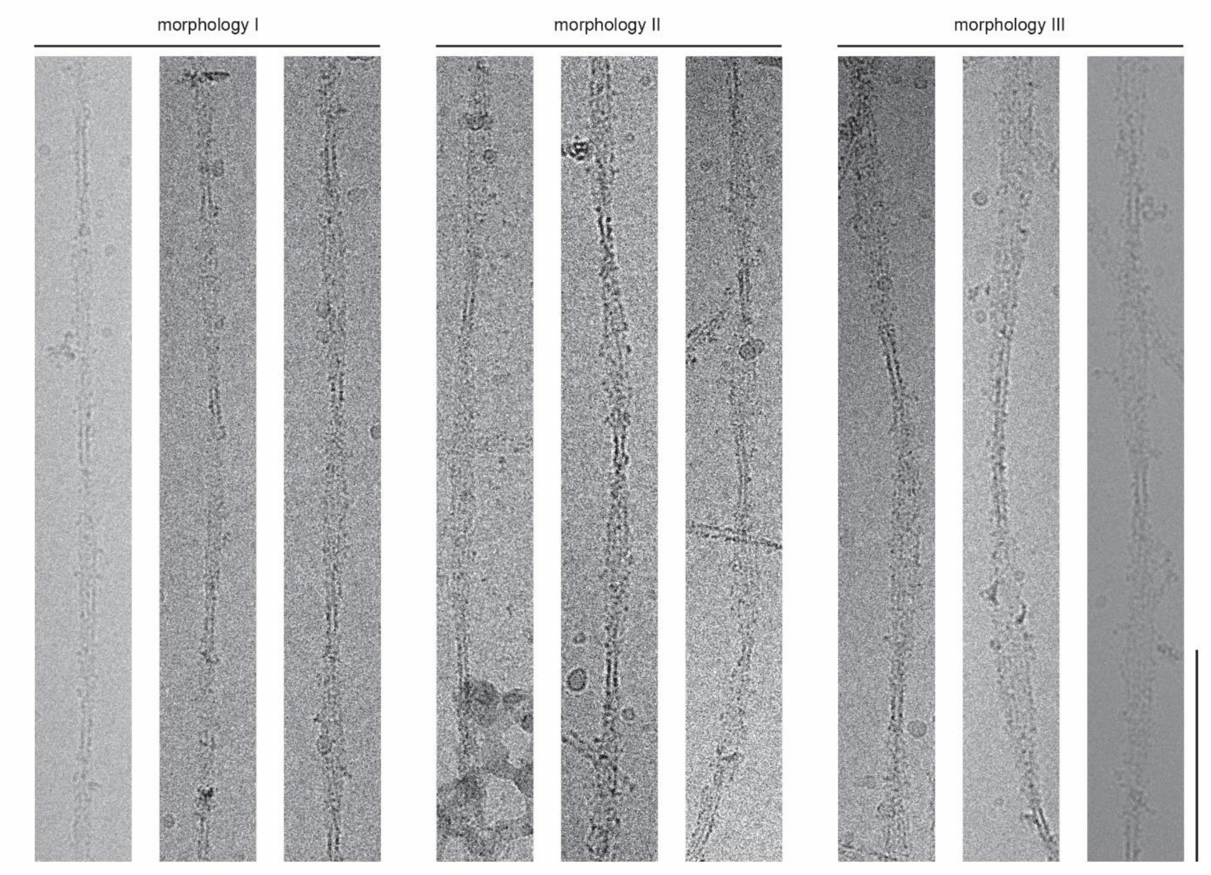

Supplementary Figure 4.

Cryo-EM images of fibril morphologies I, II and III.

Three representative images are presented for each of the three fibril morphologies. Scale bar: 100 nm

Supplementary Figure 5

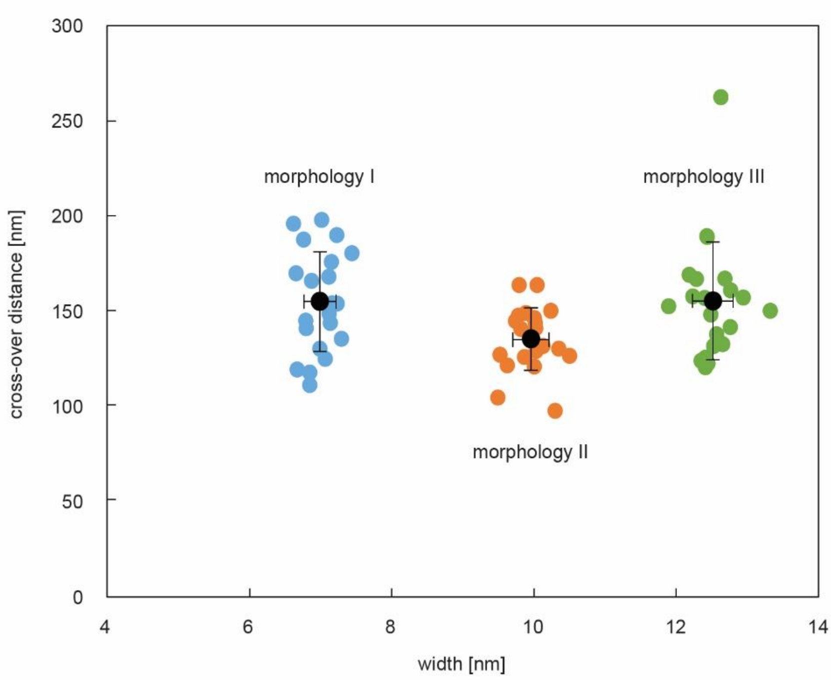

Supplementary Figure 5.

Fibril width and cross-over distances of morphologies I, II and III.

Plot of the fibril width versus cross-over distance measured with 20 fibrils of morphologies I (blue) II (orange) and III (green) each. The measurements are based on cryo-EM images. Black data points: average values. The error bars refer to the standard deviation.

Supplementary Figure 6

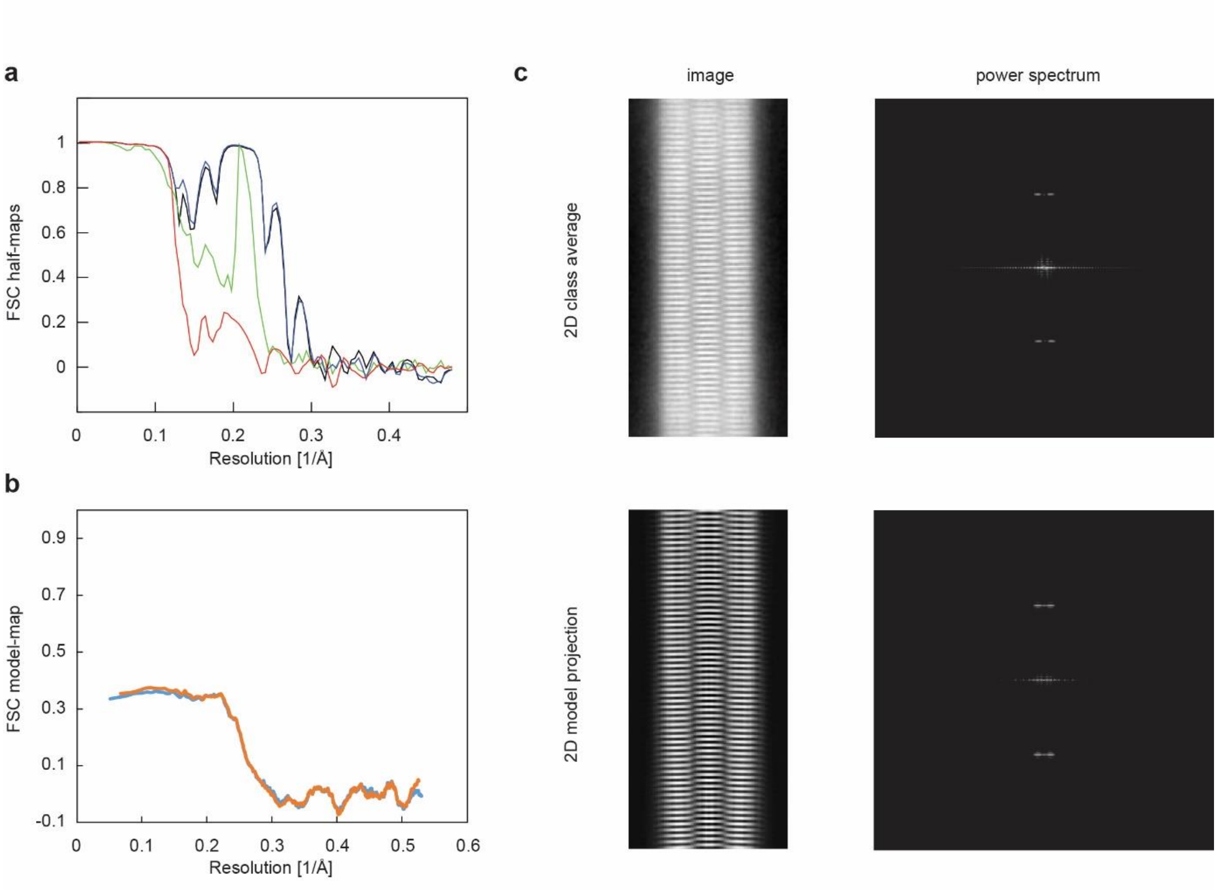

Supplementary Figure 6.

3D map of morphology I.

(a) FSC of the two half maps of the reconstruction. Black: FSC corrected; green: FSC unmasked maps; blue: FSC masked maps; red: corrected FSC phase randomized masked maps. (b) Model-map FSC. Blue: FSC unmasked maps; orange: FSC masked map. (c) Power spectrum and 2D class average of a consistent part of the fibril (left) and model density projection and power spectrum of the same fibril region (right).

Supplementary Figure 7

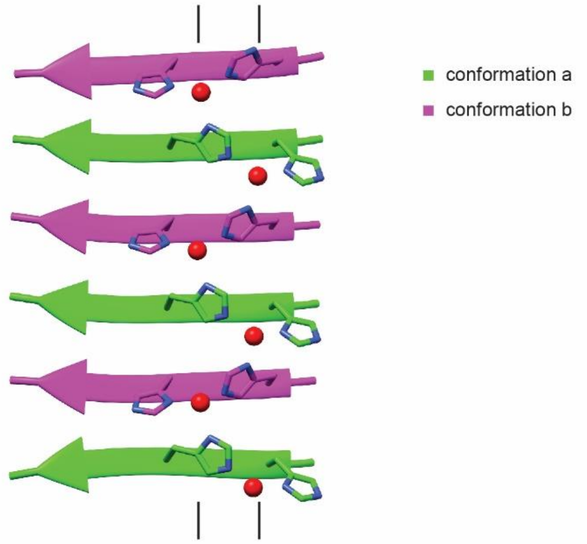

Supplementary Figure 7.

Previously described structure of an acetyl-IHVHLQI-amide cross-β sheet.

(a) Side view of the ribbon diagram of a previous NMR structure (PDB entry 5UGK) of peptide acetyl-IHVHLQI-amide [1]. The cross-β sheet is formed by two conformations (a and b) generating two rows of Zn^{2+} binding sites along the fibril axis (indicated by the vertical lines).

Supplementary Figure 8

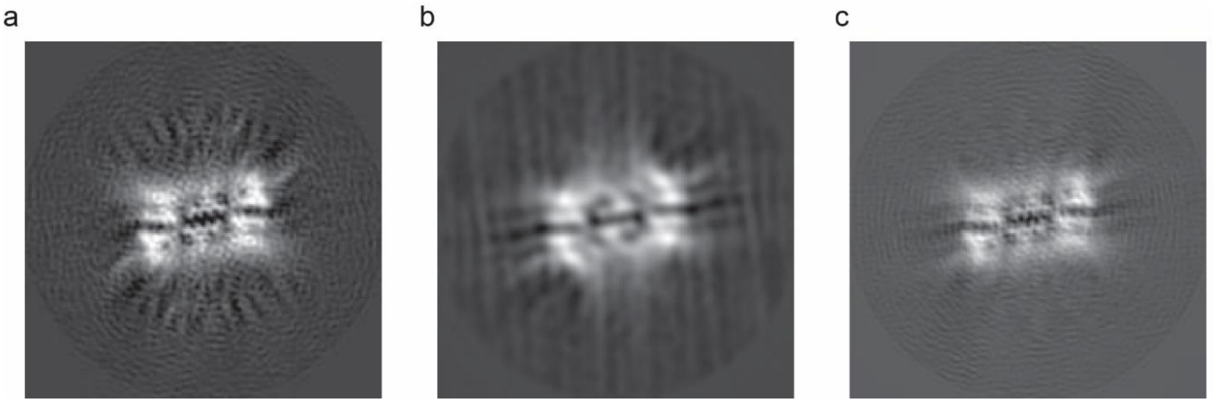

Supplementary Figure 8.

Reconstruction of morphology I with different symmetry parameters.

(a-c) 5.2 Å thick slices of the 3D maps of fibril morphology I reconstructed with a C2 symmetry and an axial repeat of 4.75 Å (a) a C2 symmetry and an axial repeat of ~9.4 Å (b) or with a pseudo 2_1 screw symmetry, combined with an axial translation of ~4.7 Å (c).

Supplementary Figure 9

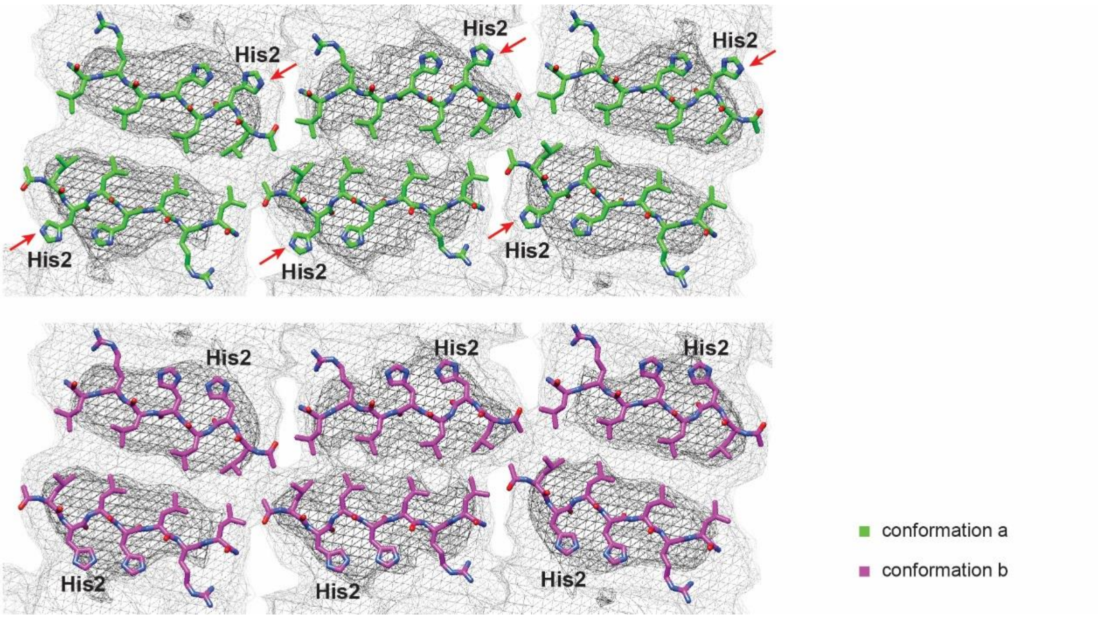

Supplementary Figure 9.

Comparison of our structure with two previously described peptide conformations.

Overlay of our 3D map of peptide acetyl-LHLHLRL-amide, which was rendered at 1σ (light grey) and 3σ (dark grey), with peptide models corresponding to conformation a (green) and b (magenta). The histidines in the conformation a do not correspond well with the density, as they protrude from it, as indicated by the red arrows.

Supplementary Figure 10

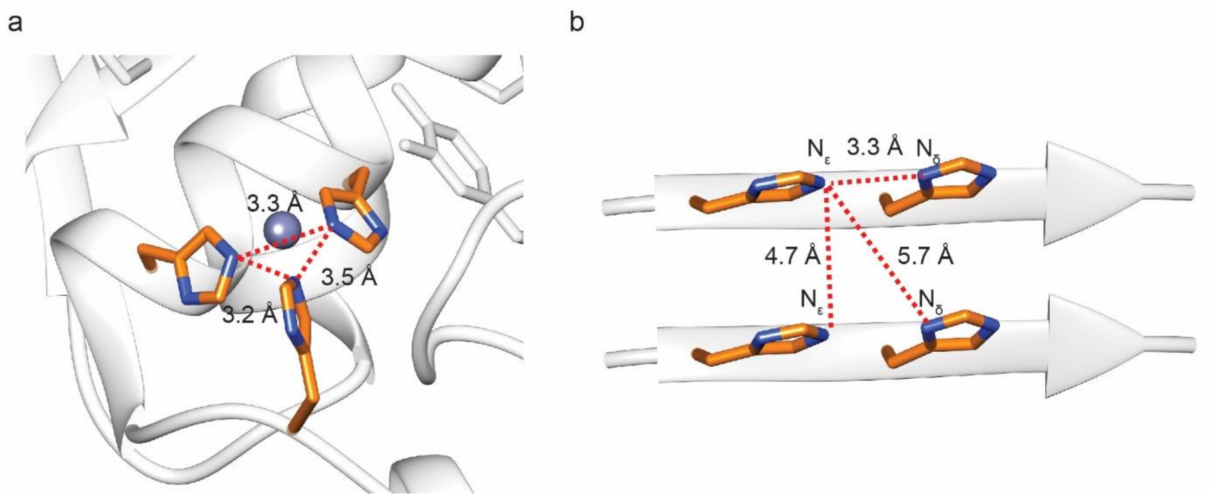

Supplementary Figure 10.

Distances between the nitrogen atoms in our fibril and in known zinc binding sites.

(a) Detailed view of the catalytic center of the human matrix metalloproteinase-13 (PDB entry 1XUD) [2], showing the distances between the zinc-binding nitrogen atoms of the three histidine residues. The zinc ion is represented as a grey sphere. (b) Distances of the histidine nitrogen atoms from neighboring strands within the same cross-β sheet of the fibril core.

Supplementary Figure 11

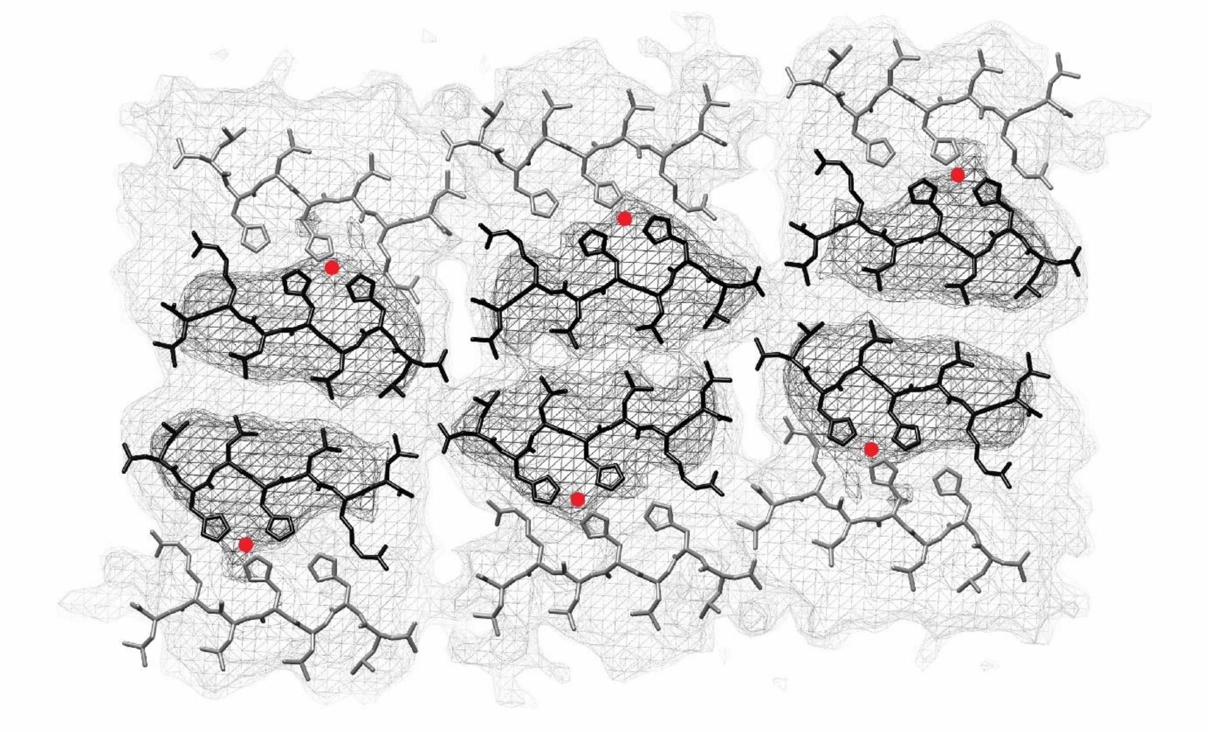

Supplementary Figure 11.

Possible features of the map that may arise from Zn^{2+} ions.

Overlay of our 3D map of peptide acetyl-LHLHLRL-amide, which was rendered at 1σ (light grey) and 3σ (dark grey), superimposed with the model of the fibril core (black sticks) and a possible placement of peptides in the outer leaflet (grey sticks) as well as the possible position of the Zn^{2+} ions (red spheres).

Supplementary Figure 12

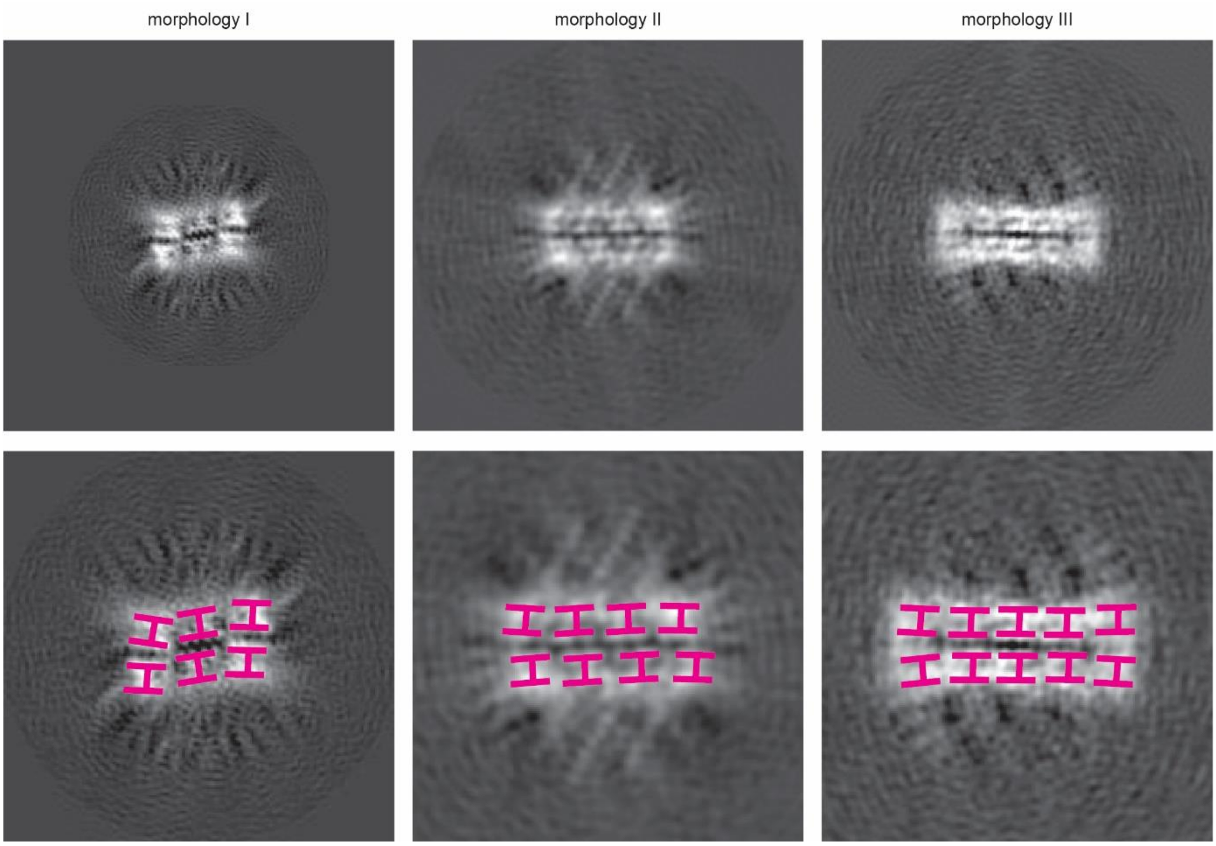

Supplementary Figure 12.

Comparison of the 3D maps of fibril morphologies I, II and III.

(Top) 5.2 Å thick slices of the 3D maps of fibril morphologies I, II and III. (Bottom) overlay of the cross-section with H-shaped features to indicate the general building blocks of the fibrils.

Supplementary Tables

Supplementary Table 1

**Microscope**	Titan Krios (Thermo Fisher Scientific)
**Camera**	K2 Summit (Gatan)
**Acceleration voltage (kV)**	300
**Magnification**	x 130,000
**Defocus range (μm)**	-0.8 to -2.0
**Dose rate (e^-/Å^2/s)**	5.3
**Number of movie frames**	40
**Exposure time (s)**	8
**Total electron dose (e^-/Å^2)**	42.7
**Pixel size (Å)**	1.04
**Gatan imaging filter**	20 eV
**Mode**	Counting mode
**Box size (pixel)**	200
**Inter box distance (Å)**	18.3
**Number of extracted segments**	38413
**Number of segments after 2D classification**	38413
**Number of segments after 3D classification**	38413
**Resolution, 0.143 FSC criterion (Å)**	3.78
**Map sharpening B-Factor (Å^2)**	-82.5
**Helical rise (Å)**	4.75
**Helical twist (°)**	-0.56
**Symmetry imposed**	C1

Supplementary Table 1.

Structural statistics of cryo-EM data collection and image processing of the major morphology of the seeded in vitro fibrils.

Supplementary Table 2

**Model resolution, 0.143 FSC criterion (Å)**	3.9
**Model composition**	
**Non-hydrogen atoms**	2010
**Protein residues**	210
**Ligands**	0
**RMSDs**	
**Bond length (Å)**	0.009
**Bond angle (°)**	2.303
**Validation**	
**Molprobity score**	0.5
**Clash score**	0
**Poor rotamers (%)**	0
**Ramachandran plot**	
**Favoured (%)**	100
**Allowed (%)**	0
**Disallowed (%)**	0
**EMRinger score**	
**z score**	7.51
**score**	5.18
**Map CC**	
**CCmask**	0.64

Supplementary Table 2.

Structural statistics of model building and refinement.

References

1. Lee, M. *et al.* Zinc-binding structure of a catalytic amyloid from solid-state NMR. *Proc Natl Acad Sci U S A* **114**, 6191–6196 (2017).

2. Wasserman, Z. R. Making a New Turn In Matrix Metalloprotease Inhibition. *Chem Biol* **12**, 143–144 (2005).
